# Recruitment of diverse community health center patients in a pragmatic weight gain prevention trial

**DOI:** 10.1017/cts.2022.475

**Published:** 2022-10-10

**Authors:** Hailey N. Miller, Miriam B. Berger, Sandy Askew, Melissa C. Kay, Miriam Chisholm, Gaurav Sirdeshmukh, Christina M. Hopkins, Ashley Brewer, Abigail DeVries, Marni Holder, Gary G. Bennett

**Affiliations:** 1 School of Nursing, Duke University, Durham, NC, USA; 2 Duke Digital Health Science Center, Duke University, Durham, NC, USA; 3 Department of Pediatrics, Duke University, Durham, NC, USA; 4 Duke Trinity College of Arts & Sciences, Durham, NC, USA; 5 Department of Psychology and Neuroscience, Duke University, Durham, NC, USA; 6 Piedmont Health Services, Inc., Chapel Hill, NC, USA; 7 Cityblock Health, Charlotte, NC, USA; 8 Department of Family Medicine, UNC-Chapel Hill School of Medicine, Chapel Hill, NC, USA

**Keywords:** Pragmatic trial, recruitment, obesity, community health center, diversity

## Abstract

**Introduction:**

Pragmatic trials are needed to establish evidence-based obesity treatment in primary care settings, particularly in community health centers (CHCs) that serve populations at heightened risk of obesity. Recruiting a representative trial sample is a critical first step to informing care for diverse communities. We described recruitment strategies utilized in a pragmatic obesity trial and assessed the sociodemographic characteristics and odds of enrollment by recruitment strategy.

**Methods:**

We analyzed data from Balance, a pragmatic trial implemented within a network of CHCs. We recruited participants via health center-based and electronic health record (EHR)-informed mail recruitment. We analyzed associations between sociodemographic characteristics and the return rate of patient authorization forms (required for participation) from EHR-informed mail recruitment. We also compared sociodemographic characteristics and randomization odds by recruitment strategy after returning authorization forms.

**Results:**

Of the individuals recruited through EHR-informed mail recruitment, females were more likely than males to return authorization forms; however, there were no differences in rates of return by preferred language (English/Spanish) or age. Females; underrepresented racial and ethnic groups; Spanish speakers; younger adults; and those with lower education levels were recruited more successfully in the health center. In contrast, their counterparts were more responsive to mail recruitment. Once authorization forms were returned, the odds of being randomized did not significantly differ by recruitment method.

**Conclusion:**

Health center-based recruitment was essential to meeting recruitment targets in a pragmatic weight gain prevention trial, specifically for Hispanic and Spanish-speaking communities. Future pragmatic trials should consider leveraging in-person recruitment for underrepresented groups in research.

## Introduction

Overweight and obesity affects over 70% of adults in the United States of America (USA) [1]. The prevalence of overweight and obesity is even higher among individuals living in socioeconomically disadvantaged communities, who are disproportionately Black and Hispanic, and who have heightened exposures to social and environmental factors that increase lifetime obesity risk [1,2]. The longer an individual experiences obesity, the more at-risk they are for serious chronic disease outcomes [3]. Without intervention, the prevalence of obesity is expected to rise and disparities will continue to persist.

Primary care facilities are an optimal setting for obesity prevention and treatment interventions given the long-standing clinician-patient relationships that can foster engagement in behavior change [4]. This is especially true for primary care settings in communities with socioeconomic challenges, in which access to affordable healthcare and evidence-based treatment and programs are often lacking [5]. However, primary care facilities remain an underutilized resource for obesity prevention and treatment. For these reasons, we implemented Balance, a pragmatic effectiveness trial that tested a digital weight gain prevention intervention within a federally qualified community health primary care setting among medically vulnerable adults with overweight or obesity [6].

Pragmatic trials portend strong dissemination potential as they provide real-world evidence focused on informing clinical practice, allowing for easier implementation of the recommendations [7]. Recruiting diverse participants for these trials is of utmost importance to ensure the generalizability of subsequent recommendations to adequately inform care [8]. However, of the pragmatic weight loss trials that are reported in the literature, few have enrolled participant populations that represent the linguistic, racial, and ethnic diversity of the USA [9,10].

Due to the paucity of literature on successful recruitment strategies for pragmatic obesity treatment research in community-based primary care, we analyzed data from the Balance trial to (1) describe recruitment strategies used for a pragmatic digital weight gain prevention trial and (2) assess the sociodemographic characteristics and odds of enrollment of groups recruited by each method.

## Materials and Methods

### Study Design

Details of the overall Balance trial design and intervention were previously described [6]. Briefly, eligible participants were randomized to standard care or a digital weight gain prevention intervention. The primary study outcome was 24-month weight gain prevention, defined as ≤3% body weight increase from baseline weight measurement. All weight measurements were extracted from the electronic health record (EHR) of the multi-site community health center organization, Piedmont Health Services (PHS). The intervention included behavior change goals with weekly self-monitoring via interactive voice response (IVR) or text (SMS); daily weighing on cellular-connected scales; responsive remote coaching from registered dietitians; and skills training. The intervention components were delivered in a pragmatic manner to ensure minimum burden on participants and PHS staff. All trial participants were required to sign a Duke Health Insurance Portability and Accountability Act (HIPAA) authorization form and verbally consent to participate prior to randomization, as described in more detail below. Trial procedures were approved by the Duke University Institutional Review Board and the PHS Board of Directors in 2016, prior to recruitment.

### Trial Setting and Population

Balance was delivered to patients of PHS, a network of 10 federally qualified community health centers in central North Carolina. PHS serves approximately 40,000 medical patients annually. To be eligible for Balance, individuals had to be PHS patients, at least 21 years of age, and receiving care at one of the five participating PHS health centers. Participating health centers were chosen based on their availability of medical nutrition therapy and proximity to the research site. In addition, patients needed to attend an outpatient medical appointment within the previous 14 days prior to enrollment with a valid weight measured and documented within the PHS EHR. Weight had to be 380 pounds or less (due to the electronic scale limitations) and a valid BMI calculated between 25.0 and 40.4 kg/m^2^ (inclusive). Participants also needed to speak English or Spanish as their primary language. To maintain a pragmatic design, participants were only excluded for safety-related reasons or for the inability to collect follow-up data from the PHS EHR. Full exclusion criteria can be found in our previously published protocol paper [6]. The target recruitment goal for Balance was 442 randomized participants, with at least 30% male and 35% Hispanic/Latino participants. Active recruitment occurred from February 2017 to December 2018.

### Recruitment Procedures

Recruitment approaches were adaptive to meet the enrollment needs of the trial, while also prioritizing a pragmatic approach. We combined health center-based recruitment and EHR-supported approaches to achieve our recruitment goals. Prior to enrollment, per privacy regulations, participants were asked to review, sign, and date a HIPAA authorization form. The authorization form included a description of the trial and an explanation of why access to their data in the EHR was necessary. In addition, the form described the protected health information (PHI) that would be collected from their record and how their information would be protected. This form granted Duke research staff permission to review their information and screen them for trial eligibility as well as to collect follow-up data from the EHR. These procedures are described in more detail below.

#### Health Center-Based Recruitment

##### Provider referral

Our initial recruitment strategy was in-person referrals by PHS providers during outpatient medical appointments. Prior to the start of recruitment, we conducted several in-person training sessions with PHS providers and staff to share and receive input on Balance’s purpose, intervention components, patient eligibility, and plans for referrals. We encouraged PHS physicians and other providers at PHS to recommend patients they thought would be a good fit for and interested in Balance during regularly scheduled visits; however, no specific rewards or incentives were provided. Aligning with a pragmatic approach, the provider could click a button in the EHR that would print out study information for a patient and the accompanying HIPAA authorization form. Lockboxes were also placed in each participating health center for Duke research staff to collect signed HIPAA forms once received from patients.

##### In-person staff recruitment

Based on feedback we received from health center providers and leaders shortly after launch, provider-led trial recruitment during appointments was onerous and thus not feasible under appointment time constraints. Therefore, upon the request of PHS, our research team deployed a bilingual (Spanish/English) research staff member to be stationed in the health center and receive provider or PHS staff referrals during appointments. If a patient was interested in Balance after speaking to a PHS provider or staff member, the patient would be referred to the research staff member. The research staff member would discuss the purpose of the study, answer questions, and provide the trial brochure and HIPAA authorization form for the patient to review and sign. The bilingual staff member was permitted to conduct recruitment and consents processes without an interpreter present.

#### EHR-informed mail recruitment

Four months into recruitment, we added EHR-informed mail recruitment to supplement in-person recruitment and to reach more potentially eligible patients. A query was applied to PHS’s EHR to identify patients who met the age and BMI criteria for Balance, as defined above, and were scheduled for an upcoming outpatient appointment. Research staff were provided an electronically secure list of names and contact information for patients identified from this method. Information about the patient’s gender, preferred language, and their upcoming appointment date and health center location was also included.

Approximately four to six weeks before their scheduled outpatient appointment, we sent recruitment materials via postal mail to patients identified in the EHR query with a valid mailing address. The mailing included a cover letter that was signed by the PHS medical director and the trial’s principal investigator and directions on directions on how to opt out of study contact, as well as a brochure with detailed information about the trial. Lastly, two copies of the HIPAA authorization form were included in the envelope –- one for the patient to sign and return in the self-addressed stamped envelope and the other to keep. Patients received materials in their preferred language (English or Spanish), based on the EHR query.

After a 10-day opt-out period ended, research staff utilized an approved phone script to call patients who had not opted out. Patients who were called were encouraged to send back the authorization form they had received in the mail and/or to discuss the trial with a study team member at the health center at their upcoming appointment. However, the number of patients called was limited by available staff time, and thus, the majority of patients did not receive a call.

#### In-person staff recruitment following mail recruitment

When feasible, our study staff would approach patients who expressed interest in the trial at their in-person appointments and had also been mailed materials, regardless of whether they were called by research staff.

#### Chart review, phone screening, and enrollment

Following receipt of the HIPAA authorization form, trained research staff conducted additional screening. Chart reviews were conducted to confirm eligibility criteria followed by phone calls to complete the eligibility screening, which included questions that could not be confirmed by the medical records. If the participant remained eligible and interested, the research staff member collected verbal informed consent and administered a short demographic survey, followed by trial randomization and onboarding.

### Analytic Approach

#### Authorization form return rates by mailed patients’ characteristics

To understand the utility of EHR-informed mail recruitment in diverse populations, we summarized the available sociodemographic characteristics – age, gender, and preferred language – of patients who were identified via the EHR and recruited through mail. We then analyzed associations between these characteristics and the authorization form return rate by patients who were mailed recruitment information.

#### Comparison of sociodemographic characteristics by recruitment method

Individuals who returned the HIPAA authorization form and enrolled in Balance were grouped by their method of recruitment: 1) Participants who were recruited in the health center (i.e., in-person staff recruitment or provider referral) and returned authorization forms in the health center; 2) Participants who were recruited via mail and returned authorization forms via mail; and 3) Participants who were recruited by both approaches and returned authorization forms in the health centers. For analyses, these groups were labeled 1) health center, 2) mail, and 3) combination, respectively. The groups’ sociodemographic characteristics were then summarized and compared using chi-square tests for categorical variables and t-tests for continuous variables. We also compared the odds of randomization after the return of authorization forms by recruitment method.

## Results

**Fig. 1.**
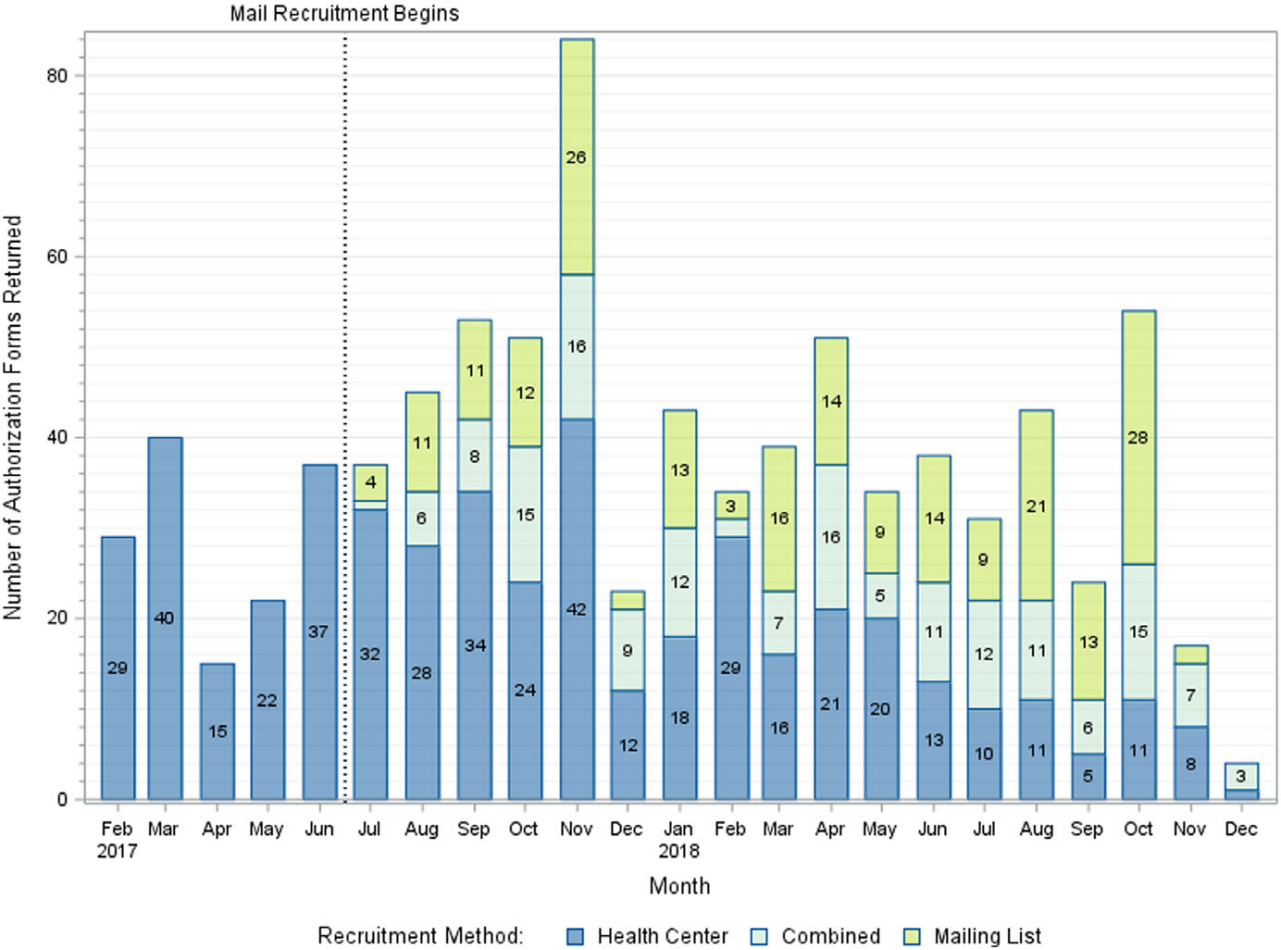
Authorization forms returned per month of the recruitment period by recruitment method.

**Fig. 2.**
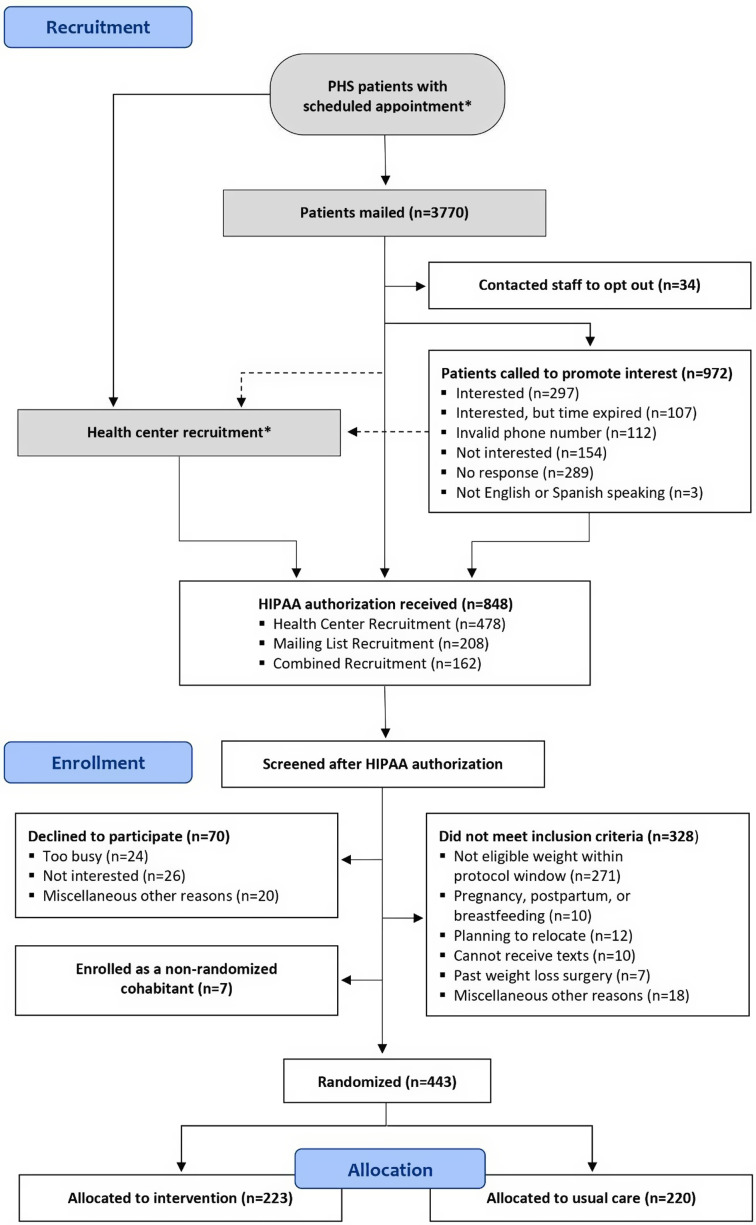
Balance recruitment and enrollment consort diagram. *The number of patients with scheduled appointments is not fully known. The number of patients informed about the study by staff and healthcare providers during appointments at the community health centers could not be quantified.

A mean of 8.7 (SD = 4.4) HIPAA authorization forms was received per week during active trial recruitment, totaling 848 authorization forms received throughout the 22.5-month recruitment period [see Fig. 1]. Among participants who returned authorization forms, 726 (86%) were eligible upon chart review and 443 (52%) were ultimately randomized – 223 to the intervention and 220 to usual care [see Fig. 2].

### Authorization Form Return Rates by Mailed Patients’ Characteristics

A total of 3,952 individuals were identified in the EHR query, the vast majority (95%) of whom had valid mailing addresses and were mailed recruitment letters [see Table 1]. Among patients who were mailed letters, a total of 34 called the study team to opt out of recruitment during the 10-day waiting period. Of the remaining patients, we attempted to call 972 (26%) and reached 70% of those patients (*n* = 680). Approximately 6% of patients returned their authorization forms from this group. Among individuals who were identified by the EHR, females were more likely than males to return authorization forms (*p* < 0.0001). There were no differences in rates of return by preferred language or age. However, there were differences in the method by which authorization forms were returned, as described below.

**Table 1.** Patient sociodemographic characteristics of patients who were mailed by the last stage of recruitment completed

	Total (*n* = 3770)	Never returned HIPAA authorization (*n* = 3400)	Lost prior to randomization (*n* = 177)	Randomized (*n* = 193)	*P*-value
**Gender, *n* (%)**					<0.0001
Female	2250	1983 (88%)	117 (5%)	150 (7%)	
Male	1348	1252 (93%)	53 (4%)	43 (3%)	
Unknown*	*172*	*165*	*7*	*0*	
**Preferred Language, n (%)**					0.25
English	2518	2230 (89%)	131 (5%)	157 (6%)	
Spanish	447	403 (90%)	25 (6%)	19 (4%)	
Unknown*	*805*	*767*	*21*	*17*	
**Age, *n*, mean (SD)**	3757	3388, 50.4 (15.6)	176, 52.3 (14.0)	193, 51.6 (13.8)	0.14

* Participants in the “unknown” category are not included in statistical tests because the demographic information available was related to the stage of recruitment by design.

### Comparison of Sociodemographic Characteristics by Recruitment Method

Participants who returned the HIPAA form (*n* = 848) were majority female (59%), English-speaking (66%), and had a mean age of 49.0 (SD = 13.7) years. Further, 24% identified as Hispanic/Latino, 13% were African American or Black, 14% were White and 46% had unknown race and ethnicity [see Table 2].

**Table 2.** Patient sociodemographic characteristics by method of recruitment in patients who returned HIPAA authorization forms

	Total (*n* = 848)	Health Center (*n* = 478)	Combined (*n* = 162)	Mail (*n* = 208)	*P*-value
**Gender, *n* (%)**					0.003
Female	499	229 (46%)	130 (26%)	140 (28%)	
Male	150	56 (37%)	30 (20%)	64 (43%)	
Unknown*	*199*	*193*	*2*	*4*	
**Race/ethnicity, *n* (%)**					<0.0001
Hispanic (of any race)	205	158 (77%)	30 (15%)	17 (8%)	
White	118	42 (36%)	22 (19%)	54 (46%)	
African American/Black	108	45 (42%)	31 (29%)	32 (30%)	
Another Race	20	10 (50%)	3 (15%)	7 (35%)	
Unknown*	*397*	*223*	*76*	*98*	
**Preferred Language, *n* (%)**					<0.0001
English	563	246 (44%)	128 (23%)	189 (34%)	
Spanish	285	232 (81%)	34 (12%)	19 (7%)	
**Education level, *n* (%)**					<0.0001
Less than high school education	84	58 (69%)	14 (17%)	12 (14%)	
High school graduate	151	94 (62%)	36 (24%)	21 (14%)	
Some college/Vocational or trade school/Associate degree	159	79 (50%)	25 (16%)	55 (35%)	
College graduate (4-year degree or beyond)	60	26 (43%)	11 (18%)	23 (38%)	
Unknown*	*394*	*221*	*76*	*97*	
**Physically active, *n* (%)**					0.009
Yes	268	137 (51%)	52 (19%)	79 (30%)	
No	181	116 (65%)	33 (18%)	32 (18%)	
Unknown*	*399*	*225*	*77*	*97*	
**Age, *n*, mean (SD)**	623	254, 44.8 (12.2)	162, 49.2 (13.6)	207, 54.0 (13.7)	<0.0001
**BMI, *n*, mean (SD)**	754	418, 32.9 (5.4)	160, 32.3 (4.3)	208, 33.6 (5.8)	0.07
**Hours of sleep per night, n, mean (SD)**	451	255, 6.9 (1.3)	85, 6.7 (1.5)	111, 6.9 (1.4)	0.54
**PHQ-2 Depression Score, n, mean (SD)**	450	254, 0.9 (1.3)	85, 1.0 (1.5)	111, 0.9 (1.1)	0.93

* Participants in the “unknown” category are not included in statistical tests because the demographic information available was related to the method of recruitment: Limited demographic information was provided to the study for participants who were mailed, but no information was available for those recruited on-site until they were screened and completed the baseline survey.

As described in Table 2, the vast majority (75%) of HIPAA forms were collected during health center-based recruitment, 19% of which were collected after forms were mailed. Among individuals who returned forms, we observed significant differences by method of recruitment for several sociodemographic characteristics, including gender, race and ethnicity, preferred language, education level, and age. Specifically, females who returned forms were most likely to be recruited at the health center (46%), while males were most likely to be recruited by mail (43%; *p* = 0.003). Adults who identified as Hispanic/Latino and/or Black/African American were most likely to be recruited at the health center. However, White adults were most likely to be recruited by mail (*p* < 0.0001). Although the most frequent recruitment method for English and Spanish-speaking adults was at the health center, a higher proportion of adults who spoke Spanish were recruited at the health center, as compared to those who spoke English (81% vs. 44%, *p* < 0.0001). In addition, the proportion of adults with some college or a college degree recruited via mail was more than twice the proportion for adults with a high school education or less (38 and 35% vs. 14%, *p* < 0.0001). Lastly, the mean age of adults recruited at the health center was 44.8 years, while adults recruited by mail or a combined approach were, on average, 54.0 and 49.2 years old, respectively (*p* < 0.0001).

There was no significant difference between recruitment groups in the odds of being randomized after an authorization form was returned [see Table 3]. As such, similar trends were observed between participant characteristics and recruitment method within the randomized subgroup of patients as were observed within the larger group of patients who returned the authorization form. This included significant differences by gender, race and ethnicity, preferred language, education level, and age [see Supplementary Table 1].

**Table 3.** Odds of patients being randomized after returning an authorization form by recruitment method (n = 848)

	*n*	Randomized (*n* = 443)	Never Randomized (*n* = 405)	OR (95% CI)	*P*- value
**Health Center**	478	250 (52%)	228 (48%)	1.02 (0.37, 1.41)	0.99
**Combined**	162	85 (52%)	77 (48%)	1.02 (0.68, 1.54)	
**Mail**	208	108 (52%)	100 (48%)	ref	

OR = Odds Ratio; CI: Confidence Interval.

## Discussion

This pragmatic trial testing a digital weight gain prevention in a primary care setting was successful at achieving the goal of recruiting and enrolling a diverse participant sample, including individuals whose preferred or only spoken language was Spanish. This required modifying initial recruitment plans and deploying trial staff to conduct in-person recruitment at the health centers.

To maintain a pragmatic approach, our first recruitment method was provider referral. However, after a few months of in-person recruitment from providers, internal PHS reports indicated that only 10% of patients who had scheduled appointments and met main eligibility criteria for Balance were referred to the trial. Provider referral is a successful recruitment strategy; however, providers have previously reported several barriers to clinical trial recruitment [11,12], with lack of time during clinical appointments to refer patients as the main obstacle [11,13,14]. This was likely a factor in Balance. Like many healthcare systems, PHS providers are allotted 20-minute primary care appointment slots; clinical trial recruitment often cannot be prioritized given the patients’ imminent and complex health and social needs. Anecdotally, some PHS providers also shared discomfort with referring a patient to the trial without adequate time to discuss all the pertinent information, including the use of patient EHR data. Another barrier that has been previously reported is providers feeling they lack sufficient knowledge about clinical trials to adequately discuss the study with their patients [11,15]. Our team attempted to mitigate this concern by providing training before recruitment launch; however, some providers who were familiar with our previous trials that focused on weight loss [16,17] expressed hesitation about the Balance’s focus on weight gain prevention. This highlights the need for provider buy-in for successful recruitment.

Thus, our team used an adaptive recruitment approach to meet trial recruitment goals. These included in-person staff recruitment at the health center and EHR-informed mail recruitment, to increase enrollment rates and alleviate the constraints of recruitment on health center providers. While recruiting in-person, we asked other trusted PHS staff, such as medical assistants, to recommend patients to Balance during their regularly scheduled visit time. To avoid interrupting care delivery and reduce burden on providers and staff, our trial staff spoke with patients themselves, allowing them to ask questions and seek clarity. This represents a promising approach, in that trusted care team members are still central to the recruitment process, but ultimately not responsible for enrolling participants. Nonetheless, this recruitment mechanism was still resource-intensive and costly, requiring our team to deploy in-person trial staff to be present on a regular basis, roughly equivalent to one full-time bilingual research coordinator.

The EHR-informed mail approach complemented in-person recruitment and did not necessitate extensive staff effort. Yet, it was not equally effective for all populations. Adults who identified as Hispanic/Latino, spoke Spanish, and/or had lower education levels were more likely to be recruited in-person, compared to EHR-informed recruitment. This was also true for individuals who identified as Black/African American. However, the differences were not as notable as EHR-informed mail recruitment was still a significant source of recruitment for this group. This supports previous literature that mail is an effective recruitment method for Black and African Americans [18].

Hispanic/Latino adults and/or those who speak Spanish are among the most underrepresented groups in clinical trials [19,20]. In fact, oftentimes, trial protocols exclude individuals who speak Spanish and/or do not provide clinical trial materials in Spanish [21,22]. Despite these barriers to clinical trial enrollment that often lead to underrepresentation of Hispanic/Latino adults, previous research indicates that they are as willing to participate in research as their non-Hispanic White counterparts [23,24]. We observed similar findings in Balance: Balance did not demonstrate differences in the rates of trial interest in my preferred language, as evidenced by the number of individuals who returned authorization forms after being mailed materials. As previously noted, however, there was a difference between English- and Spanish-speaking patients in how authorization forms were collected. This finding underscores the importance of offering multiple recruitment modalities to increase research participation among individuals from diverse backgrounds. If teams are resource-constrained, our results indicate that focusing in-person recruitment on specific underrepresented populations is optimal. Furthermore, forging in-person connections for research participation, especially with the primary care health center that is known and trusted by patients, is critical to recruitment success.

We identified several additional barriers to pragmatic clinical trial recruitment and enrollment that should be considered during trial planning. First, privacy and research regulations required a signed HIPAA authorization form prior to patient screening and enrollment by trial research staff. This was a challenge for many PHS patients who expressed interest in the trial lacked time to review and fill out forms or failed to return the forms. However, it should be noted that this barrier would not be present if this trial were implemented by community health center staff instead of external research partners. Similarly, we were challenged to reach and/or enroll patients within the 14-day window of their appointment to extract a recent baseline weight from the EHR. This ultimately resulted in 378 ineligible patients, 180 of whom had previously returned their authorization forms. In future trials, mailed recruitment information may need to be sent further in advance to allow enough time for screening and enrollment processes. Finally, because PHS attempts to serve patients even if they arrive outside of their scheduled appointment time, appointment attendance was often unpredictable. This made it difficult for our trial staff members to be present in the health center at times when there were likely to be eligible patients (e.g., avoiding times when pregnant or pediatric patients were the majority of scheduled and/or add-on patients). We mitigated this by proactively working with PHS staff to identify specific days and sites that would likely present a large pool of eligible patients. Fortunately, all of these barriers were related to processes that support the trial itself, and not treatment delivery, and therefore would not impede real-world dissemination if the treatment was found to be effective.

This study had limitations that should be considered. Similar to many recruitment campaigns, we were unable to track all in-person interactions at the health center. Therefore, we are unable to calculate recruitment yields for in-person recruitment and compare these to recruitment yields for EHR-supported recruitment. Second, we did not possess full sociodemographic data on those recruited in-person until screening and baseline surveys were completed, when randomization was largely assured. The extent and conditions of these missing data prevented us from conducting more complex analyses of recruitment success that may have been insightful. However, as noted in the results, our findings and interpretations were not changed within the randomized sample, in which full data were available. Third, there were a number of individuals who expressed interest outside of the 14-day window and thus were not screened. Their sociodemographic characteristics are unknown and could have influenced the study findings if they had been eligible. Fourth, due to staff capacity, we were unable to enroll additional Spanish-speaking adults (beyond the 35% who were in our trial), despite their interest in the study. Finally, formal cost analyses for recruitment methodology were not conducted in the current study. However, it should be noted that this is an important direction for future research which would be beneficial to future researchers proposing recruitment plans in populations traditionally underrepresented in clinical trials.

We also note several strengths of this trial. First, despite the less pragmatic approach of research staff conducting in-person recruitment at the health centers, many of the trial’s inclusion criteria, and intervention and evaluation components aligned with the pragmatic side of the Pragmatic-Explanatory Continuum Indicator Summary (PRECIS) [25]. In addition, Balance was implemented within a network of federally qualified community health centers serving a predominantly low-income patient population and reaching a group of medically vulnerable individuals with a high burden of overweight and obesity. Further, our study successfully recruited and enrolled Hispanic/Latino and Spanish-speaking adults, demonstrating recruitment approaches that effectively engage a regularly underrepresented group in research. Lastly, our study tested recruitment methods to successfully achieve recruitment goals for a pragmatic trial, while limiting burdens on the healthcare team and care delivery processes. This is an especially important consideration in non-academic settings and health centers in more rural areas where historically marginalized patients often receive care.

## Conclusion

Identifying best recruitment practices for enrolling diverse populations in pragmatic clinical trials is critical to establishing generalizable evidence-based treatment for real-world dissemination. However, maintaining a pragmatic approach during recruitment procedures can be difficult for many reasons. In our pragmatic trial for weight gain prevention within a medically vulnerable population, we found that provider referral on its own was insufficient to achieve our recruitment goals. In-person staff recruitment at participating health centers was essential to meet our recruitment goals, particularly for the Hispanic and Spanish-speaking community.

## Supplementary material

For supplementary material accompanying this paper visit https://doi.org/10.1017/cts.2022.475. click here to view supplementary material
