# Supplementary material for: Recruitment of diverse community health center patients in a pragmatic weight gain prevention trial
Source: J Clin Transl Sci. 2022 Oct 10;7(1):e22. doi: 10.1017/cts.2022.475 (PMC9879902; doi:10.1017/cts.2022.475)
Supplement: Supplementary file 1 [file S2059866122004757sup001.docx]

**Supplementary Table 1:** Patient sociodemographic characteristics by method of recruitment in patients who were randomized

|  | Total  (n=443) | On-site only  (n=250) | Both on and  off-site  (n=85) | Off-site only  (n=108) | P-value |
| --- | --- | --- | --- | --- | --- |
| Gender, n (%) |  |  |  |  | .004 |
| Female | 354 | 202 (57%) | 69 (20%) | 83 (23%) |  |
| Male | 89 | 48 (54%) | 16 (18%) | 25 (28%) |  |
| Race/ethnicity, n (%) |  |  |  |  | <.0001 |
| Hispanic (of any race) | 200 | 154 (77%) | 29 (15%) | 17 (8%) |  |
| White | 115 | 42 (37%) | 22 (19%) | 51 (44%) |  |
| African American/Black | 106 | 43 (41%) | 31 (29%) | 32 (30%) |  |
| Another Race | 19 | 9 (47%) | 3 (15%) | 7 (35%) |  |
| *Unknown** | *3* | *2* | *0* | *1* |  |
| Preferred Language, n (%) |  |  |  |  | <.0001 |
| English | 286 | 118 (41%) | 68 (24%) | 100 (35%) |  |
| Spanish | 157 | 132 (84%) | 17 (11%) | 8 (5%) |  |
| Education level, n (%) |  |  |  |  | <.0001 |
| Less than high school education | 82 | 56 (68%) | 14 (17%) | 12 (15%) |  |
| High school graduate | 149 | 93 (62%) | 35 (24%) | 21 (14%) |  |
| Some college/Vocational or  trade school/Associate degree | 156 | 78 (50%) | 25 (16%) | 53 (34%) |  |
| College graduate  (4-year degree or beyond) | 56 | 23 (41%) | 11 (20%) | 22 (39%) |  |
| Physically active, n (%) |  |  |  |  | .02 |
| Yes | 261 | 133 (51%) | 51 (20%) | 77 (29%) |  |
| No | 177 | 113 (64%) | 33 (19%) | 31 (17%) |  |
| *Unknown** | *5* | *4* | *1* | *0* |  |
| Age, n, mean (SD) | 443 | 250, 44.6 (12.1) | 85, 48.3 (13.4) | 108, 54.2 (13.4) | <.0001 |
| BMI, n, mean (SD) | 443 | 250, 32.5 (3.9) | 85, 32.6 (4.2) | 108, 33.0 (3.9) | .54 |
| Hours of sleep per night, n, mean (SD) | 443 | 248, 7.0 (1.3) | 84, 6.8 (1.5) | 108, 6.9 (1.4) | .41 |
| PHQ-2 Depression Score, n, mean (SD) | 443 | 247, 0.9 (1.3) | 84, 1.0 (1.5) | 108, 1.0 (1.1) | .91 |

*Participants in the “unknown” category are not included in statistical tests.
